# A Chinese Medicine Formula (Bushen Huoxue Tongluo) for the Treatment of Chronic Subjective Tinnitus: A Study Protocol for a Pilot, Assessor-Blinded, Randomized Clinical Trial

**DOI:** 10.3389/fphar.2022.844730

**Published:** 2022-03-30

**Authors:** Hong Wei Zhang, Kammy N. K. Yeung, Michael C. F. Tong, Zhi-Xiu Lin, Waitsz W. T. Chang, Iris H-Y. Ng, Chi Him Sum, Ka Chun Leung, Kam Leung Chan, Kit Ngan, Tie Jun Tong

**Affiliations:** ^1^ Faculty of Medicine, School of Chinese Medicine, The Chinese University of Hong Kong, Shatin, Hong Kong SAR, China; ^2^ Hong Kong Institute of Integrative Medicine, The Chinese University of Hong Kong, Shatin, Hong Kong SAR, China; ^3^ Department of Otorhinolaryngology, Head and Neck Surgery, Faculty of Medicine, The Chinese University of Hong Kong, Shatin, Hong Kong SAR, China; ^4^ Prime & Naturals Clinic, Hong Kong, Hong Kong SAR, China; ^5^ Department of Mathematics, Hong Kong Baptist University, Hong Kong, Hong Kong SAR, China

**Keywords:** Chinese medicine, tinnitus, randomized controlled trial, pilot, clinical trial protocol, herb

## Abstract

**Background:** Tinnitus is a common problem worldwide. There is still no effective method to cure it. Traditional Chinese medicine (TCM) may be a potentially effective treatment approach for tinnitus. However, there is still no clinical trial with scientifically rigorous methodology to evaluate the treatment effect of TCM for tinnitus. Therefore, we propose a pilot study to inform the feasibility of a future full-scale RCT to establish the efficacy of TCM formula for tinnitus.

**Objectives:** The aim of this study is to determine the feasibility of a full-scale RCT and explore whether a TCM formula (BHT) has an additional effect on improving tinnitus when compared to informative counseling alone.

**Design:** An assessor-blinded, randomized, controlled clinical trial is used.

**Participants:** Twenty-four patients with chronic subjective tinnitus will be enrolled.

**Interventions:** The patients will be allocated randomly to receive a TCM formula (BHT, Bushen Huoxue Tongluo) and informative counseling or informative counseling alone. The oral BHT herbal granules will be taken twice per day continuously for 8 weeks.

**Main outcome measures:** The primary outcomes include recruitment rate, intervention completion rate, and data completion rate to evaluate the feasibility. The secondary outcomes include Tinnitus Handicap Inventory, tinnitus functional index, tinnitus sensation level, self-rated visual analogue scale on tinnitus loudness and annoyance, Pittsburgh Sleep Quality Index, Hospital Anxiety and Depression Scale, and adverse event. The outcome measures will be collected at baseline, end of treatment, and 4-week follow-up.

**Discussion**: This trial is currently ongoing and is recruiting patients. The expected study results will find some preliminary evidence about the clinical effectiveness of BHT on chronic tinnitus and will also determine if it is feasible to conduct a full-scale RCT of BHT and identify the necessary changes to the protocol if possible.

**Clinical Trial Registration:** https://clinicaltrials.gov/, identifier ChiCTR2100046632.

## Introduction

Tinnitus is the perception of sound in the absence of external auditory stimulation. It is often described as a ringing, buzzing, or hissing sound in the ears or head. Tinnitus can be broadly classified into a subjective and an objective one. Objective tinnitus can be heard by others, and have its origin in vascular, muscular, skeletal, or respiratory structures (Henry et al., 2014). The vast majorities of those affected with tinnitus have a chronic subjective one, which is perceived only by the patient and not associated with an identifiable sound source (Henry et al., 2005; Tunkel et al., 2014). It is a common problem worldwide with a high prevalence. A systematic review reported in 2016 that the prevalence of tinnitus ranged from 5.1% to 42.7% globally with varied definitions (McCormack et al., 2016). It was estimated that about one in 10 adults has tinnitus in China and United States (Xu et al., 2011; Bhatt et al., 2016). The prevalence increases steadily with age, and it is more common in people aged between 40 and 70 years old (Henry et al., 2005). Chronic persistent, troublesome tinnitus can cause functional impairment in thought processing, concentration, sleep and even hearing, and also emotional disturbance, particularly anxiety and depression, all of which may substantially impact quality of life (Henry et al., 2005; Tunkel et al., 2014; Trevis et al., 2017). A survey in the United States has found that about half of the patients had discussed their problem with a physician (Bhatt et al., 2016).

The pathophysiology of tinnitus has not yet been completely understood. Tinnitus was traditionally thought to be a symptom resulting from inner ear damage; now, it is increasingly clear that tinnitus emerges from an intersection of both peripheral and central neurological pathways, contributors to which may involve medical (temporomandibular disorders and encephalitis), endocrinological, medication-related, or psychological factors (e.g., vulnerability–stress interactions) (Henry et al., 2014; Cheng et al., 2021). As no underlying cause is identified, the treatment of tinnitus is much challenging. There is still no scientifically validated medication or cure for this troublesome problem affecting millions of people (Tunkel et al., 2014). It has been reported that almost 60 different treatment modalities for tinnitus have been tried, including cognitive behavioral therapy (CBT), sound therapy, hearing aids, cochlear implants, pharmacotherapy, and acupuncture (Zenner et al., 2017). Sound therapies, such as tinnitus masking therapy, have been found to help relieve tinnitus. However, some patients may complain that they only substitute one unpleasant sound with a different one and interference with hearing (Henry et al., 2005). Antidepressants (such as the selective serotonin reuptake inhibitors), intratympanic steroids, or transcutaneous electrical nerve stimulation (TENS) have demonstrated improvement in severity of tinnitus; however, there is still insufficient robust evidence to support their application (Tunkel et al., 2014; Byun et al., 2020; Tutar et al., 2020). Now, only counseling and CBT are recommended to help patients mitigate the impact of tinnitus (Tunkel et al., 2014; Kallogjeri et al., 2017; Zenner et al., 2017).

There is still no United States Food and Drug Administration- or European Medicines Agency-approved drug on the market to treat tinnitus (Beebe et al., 2015).

Many people suffering from tinnitus seek help from complementary and alternative medicine (Enrico et al., 2007). Traditional Chinese medicine (TCM) is widely used to treat tinnitus in mainland China, Hong Kong, and other regions of East Asia. Many clinical studies on TCM treatment for tinnitus have been reported in recent decades. In Taiwan, a clinical trial on 21 patients has reported that a TCM formula Chai-Hu-Jia-Long-Gu-Mu-Li-Tang combined with Western medicine might have an additional effect on improving psychological sensation of tinnitus and sleep quality than Western medicine alone (Lin et al., 2015). In Japan, Yoku-kan-san, a TCM herbal formula, was found to cure tinnitus in 2 weeks on a patient enduring such a problem for 3 years (Okamoto et al., 2005). A systematic review in 2011 reported that the pooled analysis of 11 randomized controlled trials (RCTs) involving 1,231 patients with tinnitus suggested that TCM treatment might be more effective than Western medicines on improving the perception of tinnitus and some associated symptoms, such as headache, insomnia, dizzy, and vertigo (Wang and Zheng, 2011). Another systematic review in 2013 reported that TCM treatment combined with Western medicine showed a higher clinical effectiveness rate than Western medicine alone based on a pooled analysis of 18 RCTs (Wu and Hong, 2013). All these reviewed studies were conducted in mainland China with different TCM herbal formulae. The most popular herbs used in these formulae are Acori Tatarinowii Rhizome (Shichangpu), Puerariae Lobatae Radix (Gegen), Salviae Miltiorrhizae Radix et Rhizoma (Danshen), Astragali Radix (Huangqi), and Magnetitum (Cishi) (Wang and Zheng, 2011). Mechanism studies have indicated that TCM formulae could inhibit 5-HT (5-hydroxytryptamine)-induced RhoA/Rho kinase (ROCK) pathway, reduce pathologic damage of primary auditory cortex, or improve blood flow of meningeal microcirculation, which may contribute to the treatment of tinnitus (Zheng, 2006; Zhou et al., 2015; Wang et al., 2017). However, due to the poor reporting and some methodological flaws of these studies, no definite conclusion could be drawn on the TCM treatment effect on tinnitus.

Given the huge unmet clinical needs in the treatment of tinnitus and promising research findings of TCM treatment, it is worthwhile to further explore the TCM treatment for tinnitus. There is still no clinical trial with scientifically rigorous methodology to establish the treatment effect of TCM for tinnitus. There were three main problems in the previous studies. Firstly, there are some methodological limitations including no clear description of randomization and allocation concealment, no proper blinding, and no estimation of sample size (Wang and Zheng, 2011; Wu and Hong, 2013; Liu et al., 2016). Secondly, the TCM formula was applied without clearly defining the appropriate subset of patient population with corresponding syndrome or symptoms. According to TCM theory, a formula is generally organized to treat some syndrome under the guideline of treatment principle, which is developed based on TCM syndrome differentiation and thus aiming to manage some specific syndrome. Thirdly, validated and comprehensive outcome measures were seldom used. In mainland China, many self-developed scales or national evaluation criteria were used, which focused on evaluation of subjective feelings of patients about tinnitus and other associated symptoms (Wang and Zheng, 2011; Wu and Hong, 2013; Zhai et al., 2013). Such scales are not validated and difficult to be understood by Western medicine doctors.

We aim to evaluate the clinical efficacy and safety of TCM formula on chronic subjective tinnitus, in the hope of pursuing an effective treatment option for tinnitus. We have developed a TCM formula (BHT) for tinnitus based on previous studies and our clinical experience. An adequately powered RCT that compares TCM formula with placebo is appropriate to address the issue of efficacy; however, there is insufficient evidence to support a large full-scale RCT at present. Undertaking such a RCT raises important concerns including patients’ acceptance of TCM formula and adherence to treatment, as well as impact of TCM on the different aspects of tinnitus; furthermore, there is no previous study to provide some required parameters to design a definitive RCT. Therefore, we propose a small-scale pilot RCT to address whether a RCT of TCM formula for tinnitus is feasible with regard to (1) patient recruitment; (2) patient acceptability, (3) adherence to protocol, and (4) potential effectiveness of TCM formula. This pilot study is well justified by three main strengths on the study design. Firstly, a TCM formula BHT is developed based on the treatment principle of dredging the meridian passage and tonifying the essence of kidney, and prescribed to the subset of patients with corresponding TCM syndrome. Secondly, we will use validated and comprehensive scales to assess the impact and perception of tinnitus. Thirdly, we develop this study with scientific rigor following the CONSORT guideline extension to pilot study (Eldridge et al., 2016).

The natural course of subjective tinnitus varied considerably across individuals. The participants enrolled into clinical trials may demonstrate a small but statistically significant improvement, despite receiving no intervention (Phillips et al., 2018). A pilot randomized trial with controlled arm is more appropriate than an observatory study to provide preliminary data for further research. The results of this pilot study will inform a future full-scale RCT to establish the efficacy of TCM formula for chronic subjective tinnitus.

The study objectives are to determine the feasibility of performing a full-scale RCT for evaluating the effectiveness and safety of BHT for patients with chronic subjective tinnitus; to explore whether a TCM formula (BHT) has an additional effect on improving the impact of tinnitus in daily life and psychological function and also the perception of tinnitus after 8-week treatment and 4-week follow-up when compared to informative counseling alone; and to assess the safety of a TCM formula (BHT) in the patient population over the study duration of 12 weeks.

## Methods

### Design Overview

This pilot study is a randomized, assessor blind, two-arm, parallel superior trial. The trial design was developed conforming to Standard Protocol Items: Recommendations for Interventional Trials (SPIRIT) Guidelines (Chan et al., 2013). The study will be conducted in the Integrative Medical Centre (IMC), CUHK, and the Chinese Medicine Specialty Clinic cum Clinical Teaching and Research Centre at School of Chinese Medicine, CUHK. A total of 24 patients will be recruited and allocated randomly to receive a TCM formula (BHT) and informative counseling or informative counseling alone. The oral BHT herbal granules will be taken twice per day continuously for 8 weeks. The measures of feasibility and potential clinical effectiveness will be collected at baseline, end of the treatment (week 8), and after the follow-up (week 12). This trial will be conducted in accordance with the Declaration of Helsinki and ICH Good Clinical Practice.

### Subjects

#### Subjects Recruitment

The patients with chronic subjective tinnitus having no identified medical etiology will be recruited from the Otorhinolaryngology (ENT) Clinic in the Prince of Wales Hospital (PWH) and the Department of Otorhinolaryngology in the CUHK Medical Centre (CUHKMC), Hong Kong. Two Chinese medicine practitioners and two practicing otolaryngologists will work together to enroll eligible patients.

#### Specific Inclusion and Exclusion Criteria of Study Subjects

##### Inclusion Criteria

1) Diagnosis of unilateral or bilateral subjective continuous (24 h) tinnitus with normal hearing or bilateral/unilateral mild to moderate hearing loss, i.e., an average pure-tone audiometry threshold for 500, 1,000 and 2,000 Hz between 0 dB HL and 55 dB HL;
2) Aged between 18 and 60 years old;
3) Tinnitus duration of at least 6 months;
4) Moderate level of distress caused by tinnitus with scores above 38 on the Chinese (Cantonese) version of the Tinnitus Handicap Inventory (THI-CH) (Newman et al., 1996; Kam et al., 2009);
5) Having the diagnosis of TCM syndrome of kidney essence deficiency and stagnation of Qi and blood flowing in the meridian, which may have symptoms or signs of low back pain or general fatigue, frequent urination, and dark or pale tongue.

##### Exclusion Criteria

1) Has predisposing disease with tinnitus symptoms amenable to medical or surgical intervention;
2) Has diagnosis of pulsatile tinnitus, somatosounds, or objective tinnitus;
3) Has emotional, psychological, or psychiatric condition precluding full participation of the study;
4) Has ever received any clinical treatment for tinnitus within the previous 1 month;
5) Documented pregnant, breastfeeding, or planning pregnancy;
6) Has ever taken any Chinese herbal medicine treatment in the previous 1 month;
7) Has known history of allergy to Chinese herbal medicines;
8) Has ever taken any anti-coagulant or anti-platelet agents other than low-dose (≤81 mg per day) aspirin;
9) Has uncontrolled diabetes, hypertension or cardiac, liver, renal, cerebrovascular disease, or malignant diseases.

Subjects who meet every inclusion criterion and have none of the exclusion criteria will be eligible for inclusion in the study. Study candidates must be willing and able to provide written informed consent before entry into the study.

### Treatment Procedure

The eligible patients will be randomly allocated to receive TCM formula (BHT) granules and informative counseling or informative counseling alone. Two registered Chinese medicine practitioners (CMPs) with more than 5 years of clinical experience will be responsible for prescribing BHT to the patients. At the same time, the case history, signs and symptoms, and evaluation of tongue and pulse will be recorded with TCM diagnosis of syndrome.

#### Rationale and Composition of Chinese Herbal Formula BHT

According to Chinese medicine theory, the pathogenesis of chronic subjective tinnitus is mainly the insufficiency of essence to maintain the normal function of kidney and stagnation of Qi and blood flowing in the meridian through ear. So, the general treatment principle for tinnitus is to tonify the essence of kidney and dredge the meridian passage.

Following the treatment principle, we propose a Chinese herbal formula (Bushen Huoxue Tongluo Granules; BHT), which is modified from a classic TCM formula Er Long Zuo Ci Pill (耳聾左慈丸, in Chinese). The herbal compositions are shown in Supplementary Table S1.

Although no individualized syndrome differentiation is used in this study, this formula is designed following the core treatment strategy suitable for those patients with syndrome of kidney essence deficiency and Qi and blood stagnation, who generally have symptoms of low back pain, general fatigue, or dark or pale tongue from a Chinese medicine perspective. The prescription of BHT formula on such patients is the same as the ordinary clinical practice of Chinese medicine practitioner.

#### Preparation and Administration of BHT Granules

Concentrated Chinese medicine granules (CCMG) are used in this study, which are made from medicinal herbs by using modernized extraction and concentration technologies to replicate the traditional method of preparing medicinal decoction. All the herbal granules are manufactured by PuraPharm International (H.K.) Ltd., which is the only state-licensed CCMG brand in Hong Kong. Each granule used in BHT has been on the Hong Kong market for many years with separate wrapping and controlled quality.

The granules are used to improve the quality control elements of this study and reinforced the compliance of the participants. The pharmacy of Chinese Medicine Specialty Clinic will be responsible for preparing BHT granules into a mixture packaged in small single-dose sachets with a weight of 14.6 g. Patients in the treatment group will receive individually packaged doses of BHT, with each dose to be dissolved in warm water and consumed orally twice daily for 8 weeks.

#### Quality Control of BHT

High-performance liquid chromatography (HPLC) fingerprinting will be used for the quality control. We will choose catalpol, loganin, morroniside, paeonol, Tanshinone IIA, saikosaponin A, and puerarin (purity >98%, National Institute for the Control of Pharmaceutical and Biological Products) as reference compounds for quality control of BHT. The lab of Brain Research Center in CUHK will conduct the test.

#### Informative Counseling for all Subjects

Counseling will be provided by experienced audiologists about management in tinnitus. Subjects will be relieved about the epidemiology and nature of tinnitus. An explanation of the hearing test and tinnitus evaluation results will be explained by audiologist. Counseling will be provided at the beginning of intervention, and 4 and 8 weeks after the treatment.

The use of any other pharmaceutical agents, Chinese herbal medicines, or any other treatment approaches will be prohibited during the study period. We will provide general instructions on diet and lifestyle as usual clinical CM practice at the time of consultation. The patients will be asked to return for a clinical assessment from the CMP every 4 weeks.

### Randomization and Blinding

A computer-generated random allocation sequence through the block randomization method will be used. A research staff, with no clinical involvement in the trial, will be responsible for keeping the random allocation list and preparing sealed, opaque sequentially numbered envelopes, each containing a random number denoting the allocated treatment. After obtaining written informed consent, another research staff will open the envelopes serially, and refer the eligible patient to receive TCM formula (BHT) and informative counseling or informative counseling alone.

The outcome assessor will be kept blind at the intervention assignment during the whole study period. It is not feasible to blind doctors and patients in this study.

### Data Processing and Analysis

#### Baseline Assessment

All subjects will undergo otologic evaluation (including case history taking and otoscopic examination) and audiologic evaluation (including pure tone audiometry and tympanometry). Pure-tone audiometry (PTA) and tympanometry will be conducted to ensure that all subjects meet the inclusion criteria. Pure-tone air conduction thresholds will be measured at 250, 500, 1,000, 2,000, 3,000, 4,000, 6,000, and 8,000 Hz, and bone-conduction thresholds will be measured at 500, 1,000, 2,000, and 4,000 Hz using the standard Hughson-Westlake clinical procedure in 5-dB steps (Hughson and Westlake, 1994). High-frequency audiometry will also be performed at 10 kHz, 12.5 kHz, 16 kHz, and 20 kHz. The characteristics of tinnitus will involve measurement of parameters of self-perceived loudness in dB SL, pitch matching, and minimum masking level. Tinnitus pitch and loudness matching will be performed according to Vernon and Meikle’s procedure (Vernon and Meikle, 2003). Stimuli will be presented to the contralateral ear in cases of lateralized tinnitus, or randomly to either ear for bilateral tinnitus.

The following variables will be collected at baseline: age, gender, BMI, educational level, employment status, duration of tinnitus, percentage of tinnitus awareness during the day, and other coexisting health problems.

#### Primary Outcome Measures

The primary objective of this pilot study is to determine feasibility of a full-scale RCT. Specific outcomes include (1) recruitment rate, calculated as the percentage of eligible participants among those approached to determine if current eligibility criteria are too open or restrictive; (2) intervention completion rate, calculated as the percentage of recruited participants who complete the 8-week treatment (i.e., take ≥80% of the medicine); and (3) data completion rate, calculated as the percentage of recruited participants who complete the 8-week and 4-week follow-up assessments. The trial may be feasible if the recruitment rate ≥30%, intervention completion rate ≥80%, and data completion rate ≥80%, and not feasible if the recruitment rate <15%, intervention completion rate <70%, and data completion rate <70%. When the parameters fall between the two, it might be feasible if appropriate changes are made (Eldridge et al., 2016).

#### Secondary Outcome Measures

A comprehensive set of outcome measures is employed to assess mainly the following six domains: tinnitus percept, impact of tinnitus, co-occurring complaints, quality of life, and treatment-related outcomes (Hall et al., 2016).

1) Tinnitus Handicap Inventory in Chinese (Cantonese) version (THI-CH)

THI-CH is a 25-item self-administered questionnaire to quantify the impact of tinnitus on daily life. It has been demonstrated to be a reliable and valid measure of self-perceived tinnitus-related distress (Kam et al., 2009).

2) Tinnitus functional index in Chinese (Cantonese) version (TFI-CH)

TFI-CH is a 25-item self-administered questionnaire that assesses eight domains of tinnitus impact: intrusiveness, sense of control, cognition, sleep, auditory, relaxation, quality of life, and emotion. It has been demonstrated to be a reliable and valid measure for intake assessment and treatment-related changes (Kam et al., 2018).

3) Tinnitus sensation level

The tinnitus pitch and loudness matching will be measured according to Vernon and Meikle’s procedure (Vernon and Meikle, 2003).

4) Self-rated visual analogue scale (VAS) on tinnitus loudness and annoyance (McCormack et al., 1988)

VAS is a validated and reliable tool for measuring subjective experience in tinnitus loudness and annoyance (Vernon and Meikle, 2003). It will be measured per ear. If a subject has bilateral tinnitus, the VAS will be measured differently for each side.

5) Chinese (Cantonese) version of the Pittsburgh Sleep Quality Index (CPSQI)

The CPSQI is a self-rating questionnaire used to measure the quality and patterns of sleep in adults (Tsai et al., 2005).

6) Chinese (Cantonese) version of Hospital Anxiety and Depression Scale (HADS)

The Chinese HADS is a 14-item questionnaire designed to assess the severity of mild, even sub-syndromal degrees of anxiety and depression (Leung et al., 1999).

7) Adverse events

Adverse events will be recorded and coded to preferred terms as defined by the Medical Dictionary for Regulatory Activities (MedDRA), version 22.0.

All measurements will be taken at baseline, end of treatment (8 weeks after the first day of treatment), and after the 4-week follow-up. Kidney function will be examined before and after the treatment on those receiving BHT.

#### Statistical Analysis

The primary feasibility outcomes will be reported descriptively with percentage, mean or median, standard deviation, or 95% confidence interval. As a pilot, the study is not powered to detect a statistically significant difference between groups. The treatment effect will be assessed with mean difference between two groups and confidence intervals of 75%, 85%, and 95%. A figure showing these CIs, minimum important difference, and the null value will help assess both statistical significance and the potential for clinical significance (Bell et al., 2018). All analyses will be conducted according to the intention-to-treat principle. Last observation carried forward will be used for imputing missing data because tinnitus is a chronic and constant symptom. Descriptive statistics will be computed for each of the analyzed variables. Exploratory inferential analysis will be performed using repeated-measures ANCOVA. Mean differences from baseline to end of treatment in each group will be examined by paired *t*-test or Wilcoxon signed rank test. Subgroup analysis on those with or without hearing loss, and those with unilateral or bilateral tinnitus will be made. All statistical tests will be two-sided, and *p* < 0.05 is considered statistically significant. The statistical software of SPSS 23.0 will be used for analysis.

Adverse events will be categorized and percentage of those experiencing some adverse events and serious adverse events will be documented. Chi-square tests will be performed to examine differences in the proportion of total and categories of adverse events within each group.

It is anticipated to find some preliminary evidence about the clinical effectiveness of BHT on chronic tinnitus, to identify if it is feasible to conduct a full-scale RCT of BHT and necessary changes to the protocol if possible. Statistical comparisons between groups may be misleading due to the many potential problems arising from small samples and low power. The results from statistical comparisons will be treated with great caution, and reported only with explicit reference to statistical power.

#### Sample Size Estimation

As an exploratory pilot study, the stepped rules of thumb is used for estimating the pilot sample size (Whitehead et al., 2016; Bell et al., 2018). Although there is no previous study on BHT, we base the estimation of standardized effect size on two previous studies of compound TCM formula on chronic tinnitus (Lin et al., 2015; Chen et al., 2018). Using a mean difference estimate of eight and an estimate of standard deviation of 11–20 in similar populations, the standardized effect size is about 0.4 to 0.7. Assuming a reasonably medium (0.3–0.7) standardized effect size, a sample size of 10 per arm for the pilot is recommended for a 90% powered main trial. Therefore, a total of 24 patients will be needed in this trial, considering a 20% dropout rate.

### Study Monitoring and Quality Assurance

The study will be undertaken in accordance with this clinical trial protocol, ICH guidelines for Good Clinical Practice, the Declaration of Helsinki, and the applicable regulatory requirements of Hong Kong. Case report forms (CRFs) will be developed for recording the individual patient data. The investigators will assure that all reported trial data are accurate, complete, and verifiable from source documents. Work manual and training workshop will be provided to all involved research staff.

The study committee will hold regular conferences to monitor the patients’ safety and the integrity of the data with respect to the original study design, and to provide advice on study conduct. Authorized representatives of the ethics committee may visit the research clinic to perform inspections.

## Discussion

Although various Chinese herbal medicine formulae have been used to treat tinnitus in East Asia, none of them was supported by sound clinical evidence. We aim to establish a robust evidence for the clinical application of CHM formulae for chronic tinnitus. This pilot study is the first step to explore the feasibility and scientificity of a further full-scale RCT. Its expected results will help us decide on the appropriate approaches to find potential participants, the inclusion criteria, and efficient ways to manage the study and reduce the withdrawal rate. The results on the estimations of study effects will be used as a reference for deciding on the planning of a further full-scale RCT.

According to Chinese medicine theory, the pathogenesis of chronic subjective tinnitus is mainly the insufficiency of essence to maintain the normal function of kidney and stagnation of Qi and blood flowing in the meridian through ear. A literature review on the clinical studies of Chinese herbal medicines for tinnitus from 2003 to 2013 has found that the most common syndromes are insufficiency of kidney essence and stagnation of Qi and blood flowing (Qiu et al., 2015). Thus, the general treatment principle for chronic subjective tinnitus is to tonify the essence of kidney, promote blood flowing, and dredge the meridian passage around ears, Bushen Huoxue Tongluo in Chinese. The CHM formulae with such effects are widely used to treat chronic tinnitus. Studies have shown that they could effectively reduce pathological damage to the primary auditory cortex (Wang et al., 2017), improve the microcirculation of the inner ear and have a sedation effect (Zhong et al., 2001; Zheng 2006), and inhibit 5-HT (hydroxytryptamine)/Rho-associated kinase signaling pathway (Zhou et al., 2015).

Er Long Zuo Ci Pill (耳聾左慈丸 in Chinese), a traditionally well-known formula, is widely used to treat tinnitus and deafness in China. It was found to be one of the most often used formulae to treat tinnitus in the recent 30 years (Zhang et al., 2015). Some clinical trial studies have reported that Er Long Zuo Ci Pill could reduce the tinnitus loudness and hearing threshold and relieve the associated symptoms (Zhang et al., 2015; Zhang, 2017). Mechanism studies have indicated that Er Long Zuo Ci Pill could protect the cochlear hair cells from gentamicin-induced ototoxicity (Dong et al., 2010); decrease auditory brainstem response threshold; inhibit the expression of tumor necrosis factor alpha, interleukin-6, and interleukin-1 beta (Li and Wang, 2015); upregulate protein expression of aquaporin-4 in cochlear tissues (Lv et al., 2015); and reduce spontaneous discharge of external cortex of inferior colliculus and secondary auditory cortex induced by salicylic acid (Wang et al., 2009).

Er Long Zuo Ci Pill is composed of eight herbs, namely, *Rehmannia glutinosa* (Gaertn.) DC (Orobanchaceae) (Dihuang), Cornus officinalis Siebold & Zucc (Cornaceae) (Shanzhuyu), Paeonia × suffruticosa Andrews (Paeoniaceae) (Mudanpi), Bupleurum falcatum L (Apiaceae) (Chaihu), Poria cocos (Schw.) Wolf (Fuling), Magnetitum (Cishi), *Dioscorea oppositifolia* L (Dioscoreaceae) (Shanyao), and Alisma plantago-aquatica subsp. orientale (Sam.) Sam (Alismataceae) (Zexie), which has the main effect of tonifying kidney essence. To enhance its treatment effect on chronic tinnitus, some formulae modified from Er Long Zuo Ci Pill have been studied in clinical trials. It has been demonstrated that its clinical effect on tinnitus could be augmented by combining herbs with an effect of activating blood circulation (Xie et al., 2016).

Following the treatment principle, we further combine Er Long Zuo Ci Pill with several other herbs with an effect of activating blood circulation, dredging the meridians, and tranquilizing the mind to make the formula BHT. In BHT, *Rehmannia glutinosa* (Gaertn.) DC (Orobanchaceae] (Dihuang), Cornus officinalis Siebold & Zucc (Cornaceae) (Shanzhuyu), and Dipsacus asper Wall. ex DC (Caprifoliaceae) (Xuduan) have the effect of tonifying kidney essence. Paeonia × suffruticosa Andrews (Paeoniaceae) (Mudanpi), Salvia miltiorrhiza Bunge (Lamiaceae) (Danshen), Conioselinum anthriscoides “Chuanxiong” (Apiaceae) (Chuanxiong), and Prunus persica (L.) Batsch (Rosaceae) (Taoren) have the effect of activating blood circulation. Poria cocos (Schw.) Wolf (Fuling), Bupleurum falcatum L (Apiaceae) (Chaihu), Pinellia ternata (Thunb.) Makino (Araceae) (Fabanxia), Pueraria *montana* var. lobata (Willd.) Maesen & S.M.Almeida ex Sanjappa & Predeep (Fabaceae) (Gegen), and Acorus calamus var. angustatus Besser (Acoraceae) (Shichangpu) have the effect of dredging the meridians. Ziziphus jujuba Mill (Rhamnaceae) (Suanzaoren), Magnetitum (Cishi), and Margaritifera Concha (Zhenzhumu) have the effect of suppressing hyperactive liver yang and tranquilizing the mind.

BHT is developed for those with the most common TCM syndrome, kidney essence deficiency, and stagnation of Qi and blood flowing in the meridian around the ears, in patients with chronic tinnitus. From the view of TCM, BHT is of generally neutral nature (not hot or cold), which enlarges the scope of its clinical application. During the study, the clinical symptoms and signs, pulse, and tongue diagnosis will be collected. Further analysis of its effects on those patients with TCM diagnosis of cold, warm, or cold–heat complication will be made.

The study results will provide an estimation of the general add-on treatment effect of CHM on chronic tinnitus. The choice of informative counseling alone as control will help to control some non-specific treatment effects, such as natural history and regression to the mean; however, it should be noted that the placebo effect could not be ruled out from the comparison results. The possible placebo effect of CHM may have effects on the outcomes, especially subjective ones. A placebo-control and some objective outcomes, such as functional near-infrared neuroimaging (fNIR), may be employed in the design of future full-scale RCT.

This pilot study will provide the United States a solid basis to further design a full-scale RCT of CHM for chronic tinnitus, and also further explore the scope of clinical application of BHT and possible ways to enhance the clinical effects.

## References

[B1] Beebe Palumbo D. Joos K. De Ridder D. Vanneste S. (2015). The Management and Outcomes of Pharmacological Treatments for Tinnitus. Curr. Neuropharmacol 13 (5), 692–700. 10.2174/1570159x13666150415002743 26467416 PMC4761638

[B2] Bell M. L. Whitehead A. L. Julious S. A. (2018). Guidance for Using Pilot Studies to Inform the Design of Intervention Trials with Continuous Outcomes. Clin. Epidemiol. 10, 153–157. 10.2147/CLEP.S146397 29403314 PMC5779280

[B3] Bhatt J. M. Lin H. W. Bhattacharyya N. (2016). Prevalence, Severity, Exposures, and Treatment Patterns of Tinnitus in the United States. JAMA Otolaryngol. Head Neck Surg. 142 (10), 959–965. 10.1001/jamaoto.2016.1700 27441392 PMC5812683

[B4] Byun Y. J. Lee J. A. Nguyen S. A. Rizk H. G. Meyer T. A. Lambert P. R. (2020). Transcutaneous Electrical Nerve Stimulation for Treatment of Tinnitus: A Systematic Review and Meta-Analysis. Otol Neurotol 41 (7), e767–e775. 10.1097/MAO.0000000000002712 32472915

[B5] Chan A. W. Tetzlaff J. M. Altman D. G. Laupacis A. Gøtzsche P. C. Krleža-Jerić K. (2013). SPIRIT 2013 Statement: Defining Standard Protocol Items for Clinical Trials. Ann. Intern. Med. 158 (3), 200–207. 10.7326/0003-4819-158-3-201302050-00583 23295957 PMC5114123

[B6] Chen S. Tan X. Fei L. Xiang X. (2018). Clinical Observation on Idiopathic Tinnitus Treated with Acupuncture, Buzhong Yiqi Tang and Cizhu Wan. Zhongguo Zhen Jiu 38 (4), 369–373. Chinese. 10.13703/j.0255-2930.2018.04.007 29696920

[B7] Cheng Y. F. Xirasagar S. Kuo N. W. Chung S. D. Lin H. C. (2021). Association of Erectile Dysfunction with Tinnitus: a Nationwide Population-Based Study. Sci. Rep. 11, 6982. 10.1038/s41598-021-86441-6 33772046 PMC7997891

[B8] Dong Y. Cao B. Y. Wang J. Ding D. L. Han Z. F. Shi J. R. (2010). Effects of Erlong Zuoci Pill and its Disassembled Prescriptions on Gentamicin-Induced Ototoxicity Model *In Vitro* . Chin. J. Integr. Med. 16 (3), 258–263. Chinese. 10.1007/s11655-010-0258-x 20694782

[B9] Eldridge S. M. Chan C. L. Campbell M. J. Bond C. M. Hopewell S. Thabane L. (2016). CONSORT 2010 Statement: Extension to Randomised Pilot and Feasibility Trials. BMJ 355, i5239. 10.1136/bmj.i5239 27777223 PMC5076380

[B10] Enrico P. Sirca D. Mereu M. (2007). Antioxidants, Minerals, Vitamins, and Herbal Remedies in Tinnitus Therapy. Prog. Brain Res. 166, 323–330. 10.1016/S0079-6123(07)66029-4 17956795

[B11] Hall D. A. Haider H. Szczepek A. J. Lau P. Rabau S. Jones-Diette J. (2016). Systematic Review of Outcome Domains and Instruments Used in Clinical Trials of Tinnitus Treatments in Adults. Trials 17 (1), 270. 10.1186/s13063-016-1399-9 27250987 PMC4888312

[B12] Henry J. A. Dennis K. C. Schechter M. A. (2005). General Review of Tinnitus: Prevalence, Mechanisms, Effects, and Management. J. Speech Lang. Hear. Res. 48 (5), 1204–1235. 10.1044/1092-4388(2005/084) 16411806

[B13] Henry J. A. Roberts L. E. Caspary D. M. Theodoroff S. M. Salvi R. J. (2014). Underlying Mechanisms of Tinnitus: Review and Clinical Implications. J. Am. Acad. Audiol. 25 (1), 5126–126. 10.3766/jaaa.25.1.2 PMC5063499 24622858

[B14] Hughson W. Westlake H. (1994). Manual for Program Outline for Rehabilitation of Aural Casualties Both Military and Civilian. Trans. Am. Acad. Ophthalmol. Otolaryngol. 48 (Suppl. l), 1–15.

[B15] Kallogjeri D. Piccirillo J. F. Spitznagel E. Hale S. Nicklaus J. E. Hardin F. M. (2017). Cognitive Training for Adults with Bothersome Tinnitus: A Randomized Clinical Trial. JAMA Otolaryngol. Head Neck Surg. 143 (5), 443–451. 10.1001/jamaoto.2016.3779 28114646 PMC5824313

[B16] Kam A. C. Cheung A. P. Chan P. Y. Leung E. K. Wong T. K. van Hasselt C. A. (2009). Psychometric Properties of the Chinese (Cantonese) Tinnitus Handicap Inventory. Clin. Otolaryngol. 34 (4), 309–315. 10.1111/j.1749-4486.2009.01946.x 19673977

[B17] Kam A. C. S. Leung E. K. S. Chan P. Y. B. Tong M. C. F. (2018). Cross-cultural Adaptation and Psychometric Properties of the Chinese Tinnitus Functional index. Int. J. Audiol. 57 (2), 91–97. 10.1080/14992027.2017.1375162 28918676

[B18] Leung C. M. Wing Y. K. Kwong P. K. Lo A. Shum K. (1999). Validation of the Chinese-Cantonese Version of the Hospital Anxiety and Depression Scale and Comparison with the Hamilton Rating Scale of Depression. Acta Psychiatr. Scand. 100 (6), 456–461. 10.1111/j.1600-0447.1999.tb10897.x 10626925

[B19] Li Y. Wang Z. (2015). [Effect of a Randomized Controlled Trial of Erlongzuoci Pill on Expression of Senile Deafness in Mice Serum TNF-Alpha, IL-1 Beta, IL-6. [Chinese] J. Pract. Traditional Chin. Intern. Med. 29 (12), 157–159. Chinese.

[B20] Lin C. H. Kuo C. E. Yu H. C. Lai Y. K. Huang Y. C. Tsai M. Y. (2015). Efficacy of Adjuvant Chinese Herbal Formula Treatment for Chronic Tinnitus: a Retrospective Observational Study. COMPLEMENT. THER. MED. 23 (2), 226–232. 10.1016/j.ctim.2015.01.002 25847560

[B21] Liu F. Han X. Li Y. Yu S. (2016). Acupuncture in the Treatment of Tinnitus: a Systematic Review and Meta-Analysis. Eur. Arch. Otorhinolaryngol. 273 (2), 285–294. 10.1007/s00405-014-3341-7 25344063

[B22] Lv Y. Wang Z. Ma X. Zhao J. (2015). Effects of Erlong Zuoci Pills on Expression of AQP4 in Cochlear Tissue of Mice with Elderly Kidney Deficiency Deafness. Chin. J. Inf. TCM 22 (5), 69–71. Chinese.

[B23] McCormack A. Edmondson-Jones M. Somerset S. Hall D. (2016). A Systematic Review of the Reporting of Tinnitus Prevalence and Severity. Hear. Res. 337, 70–79. 10.1016/j.heares.2016.05.009 27246985

[B24] McCormack H. M. Horne D. J. Sheather S. (1988). Clinical Applications of Visual Analogue Scales: a Critical Review. Psychol. Med. 18 (4), 1007–1019. 10.1017/s0033291700009934 3078045

[B25] Newman C. W. Jacobson G. P. Spitzer J. B. (1996). Development of the Tinnitus Handicap Inventory. Arch. Otolaryngol. Head Neck Surg. 122 (2), 143–148. 10.1001/archotol.1996.01890140029007 8630207

[B26] Okamoto H. Okami T. Ikeda M. Takeuchi T. (2005). Effects of Yoku-Kan-San on Undifferentiated Somatoform Disorder with Tinnitus. Eur. Psychiatry 20 (1), 74–75. 10.1016/j.eurpsy.2004.09.034 15642449

[B27] Phillips J. S. McFerran D. J. Hall D. A. Hoare D. J. (2018). The Natural History of Subjective Tinnitus in Adults: A Systematic Review and Meta-Analysis of No-Intervention Periods in Controlled Trials. Laryngoscope 128 (1), 217–227. 10.1002/lary.26607 28425615

[B28] Qiu X. D. Ma R. H. Ding M. H. (2015). Literature Review (2003-2013) on Traditional Chinese Medicine in Syndrome Differentiation and Treatment of Tinnitus. World J. Integrated Traditional West. Med. 10 (12), 1643–1646. Chinese.

[B29] Trevis K. J. McLachlan N. M. Wilson S. J. (2017). A Systematic Review and Meta-Analysis of Psychological Functioning in Chronic Tinnitus. Clin. Psychol. Rev. 60, 62–86. 10.1016/j.cpr.2017.12.006 29366511

[B30] Tsai P. S. Wang S. Y. Wang M. Y. Su C. T. Yang T. T. Huang C. J. (2005). Psychometric Evaluation of the Chinese Version of the Pittsburgh Sleep Quality Index (CPSQI) in Primary Insomnia and Control Subjects. Qual. Life Res. 14 (8), 1943–1952. 10.1007/s11136-005-4346-x 16155782

[B31] Tunkel D. E. Bauer C. A. Sun G. H. Rosenfeld R. M. Chandrasekhar S. S. Cunningham E. R. (2014). Clinical Practice Guideline: Tinnitus Executive Summary. Otolaryngol. Head Neck Surg. 151 (2 Suppl. l), 533–541. 10.1177/0194599814547475 25274374

[B32] Tutar B. Atar S. Berkiten G. Üstün O. Kumral T. L. Uyar Y. (2020). The Effect of Transcutaneous Electrical Nerve Stimulation (TENS) on Chronic Subjective Tinnitus. Am. J. Otolaryngol. 41 (1), 102326. 10.1016/j.amjoto.2019.102326 31732303

[B33] Vernon J. A. Meikle M. B. (2003). Tinnitus: Clinical Measurement. Otolaryngol. Clin. North. Am. 36 (2), 293–vi. 10.1016/s0030-6665(02)00162-7 12856298

[B34] Wang R. Pi L. Chen H. Zhang H. (2017). Effects of Bushen Huoxue Recipe Combined with Neural Stem Cell Transplantation in Rats with Tinnitus. Chin. J. Tissue Eng. Res. 21 (5), 730–735. Chinese.

[B35] Wang S. Zheng R. (2011). [A Meta-Analysis on Treatment of Tinnitus with Traditional Chinese Medicine and its Medication Rules. J. Anhui TCM Coll. 30 (1), 13–16. Chinese.

[B36] Wang Y. M. Song H. Y. Tong Z. Qian S. J. Guo R. X. Jing Z. J. (2009). Effects of Er-Long-Zuo-Ci-Wan on the Spontaneous Activities of Auditory central Nucleus in Rat Model of Tinnitus Induced by Salicylate Acid. Zhongguo Ying Yong Sheng Li Xue Za Zhi 25 (3), 397–401. Chinese. 21155246

[B37] Whitehead A. L. Julious S. A. Cooper C. L. Campbell M. J. (2016). Estimating the Sample Size for a Pilot Randomised Trial to Minimise the Overall Trial Sample Size for the External Pilot and Main Trial for a Continuous Outcome Variable. Stat. Methods Med. Res. 25 (3), 1057–1073. 10.1177/0962280215588241 26092476 PMC4876429

[B38] Wu H. Hong Q. (2013). Meta-analysis on Integrated Traditional Chinese and Western Medicine in Treatment of Tinnitus. Chin. Arch. Traditional Chin. Med. 31 (3), 671–673. Chinese.

[B47] Xie X. (2016). Analysis of Curative Effect of Erlongzuoci Pill Combined With Songlingxuemaikang Capsule in the Treatment of Tinnitus. China Health Standard Management 7 (18), 127–128. Chinese.

[B39] Xu X. Bu X. Zhou L. Xing G. Liu C. Wang D. (2011). An Epidemiologic Study of Tinnitus in a Population in Jiangsu Province, China. J. Am. Acad. Audiol. 22 (9), 578–585. 10.3766/jaaa.22.9.3 22192603

[B40] Zenner H. P. Delb W. Kröner-Herwig B. Jäger B. Peroz I. Hesse G. (2017). A Multidisciplinary Systematic Review of the Treatment for Chronic Idiopathic Tinnitus. Eur. Arch. Otorhinolaryngol. 274 (5), 2079–2091. 10.1007/s00405-016-4401-y 27995315

[B41] Zhai S. Fang Y. Yang W. Gu R. Han D. Yang S. (2013). Clinical Investigation on the Beneficial Effects of the Chinese Medicinal Herb Gushen Pian on Sensorineural Deafness and Tinnitus. CELL Biochem. Biophys. 67 (2), 785–793. 10.1007/s12013-013-9536-5 23516092

[B42] Zhang D. Q. Ma W. H. Mei Z. G. (2015). Rule of Traditional Chinese Medicine in Treating Tinnitus Based on Literature and Logistic Multiple Regression Analysis. Chin. J. Inf. TCM 22 (2), 34–37. Chinese.

[B43] Zhang Q. K. Y. (2017). The Clinical Analysis of Compound Erlongzuoci Pill Combined with Auricular point Stick in and Vitamin B12 Acupoint Injection Therapy on Nervous Tinnitus of Middle and Old Aged. Chin. J. Biochem. Pharmaceuticals 37 (7), 131–133. Chinese.

[B44] Zheng G. (2006). The Microcirculation Mechanism of Xuanfu Theory and Jia Wei Tong Qi San in Removing Obstacles from Xuanfu. Chin. Archive Traditional Chin. Med. 24 (1), 18–20. Chinese.

[B45] Zhong Q. Wu R. Z. Feng Z. R. Zhang C. F. Xiao S. H. Qin D. L. (2001). Pharmacodynamics Study on the Ermingxiao Oral Liquid. Shanxi J. TCM 17 (4), 51–53. In Chinese.

[B46] Zhou C. Bi Y. Yuan X. Zhou L. (2015). Clinical Efficacy and Mechanism on Acupuncture Therapy Added Conventional Pharmacotherapy in the Treatment of Tinnitus. J. Basic Chin. Med. 21 (5), 581–587. Chinese.

