# Corrigendum: A Chinese medicine formula (Bushen Huoxue Tongluo) for the treatment of chronic subjective tinnitus: a study protocol for a pilot, assessor-blinded, randomized clinical trial

**DOI:** 10.3389/fphar.2023.1258855

**Published:** 2023-09-07

**Authors:** Hong Wei Zhang, Kammy N. K. Yeung, Michael C. F. Tong, Zhi-Xiu Lin, Waitsz W. T. Chang, Iris H-Y. Ng, Chi Him Sum, Ka Chun Leung, Kam Leung Chan, Kit Ngan, Tie Jun Tong

**Affiliations:** ^1^ Faculty of Medicine, School of Chinese Medicine, The Chinese University of Hong Kong, Shatin, Hong Kong SAR, China; ^2^ Hong Kong Institute of Integrative Medicine, The Chinese University of Hong Kong, Shatin, Hong Kong SAR, China; ^3^ Department of Otorhinolaryngology Head and Neck Surgery, Faculty of Medicine, The Chinese University of Hong Kong, Shatin, Hong Kong SAR, China; ^4^ Prime & Naturals Clinic, Hong Kong, Hong Kong SAR, China; ^5^ Department of Mathematics, Hong Kong Baptist University, Hong Kong, Hong Kong SAR, China

**Keywords:** Chinese medicine, tinnitus, randomized controlled trial, pilot, clinical trial protocol, herb

In the published article, there was an error in the **Funding** statement. This study is supported by Health and Medical Research Fund (HMRF), Food and Health Bureau, Hong Kong, China (No. 18190691).The correct **Funding** statement appears below.

“This study is supported by Health and Medical Research Fund (HMRF), Food and Health Bureau, Hong Kong, China (No. 17180941)”.

The authors apologize for this error and state that this does not change the scientific conclusions of the article in any way. The original article has been updated.

