# Supplementary material for: A Chinese Medicine Formula (Bushen Huoxue Tongluo) for the Treatment of Chronic Subjective Tinnitus: A Study Protocol for a Pilot, Assessor-Blinded, Randomized Clinical Trial
Source: Front Pharmacol. 2022 Mar 30;13:844730. doi: 10.3389/fphar.2022.844730 (PMC9006145; doi:10.3389/fphar.2022.844730)
Supplement: Supplementary file 1 [file Table1.DOCX]

**Table 1 Ingredients of the Chinese herbal formula BHT**

| **Constituent herb** | **Gram/**  **Day, herb** | **Gram/**  **Day, granule** | **Role in formula** |
| --- | --- | --- | --- |
| Rehmanniae Radix (Dihuang, 地黃) | 12 | 2.4 | Sovereign (Jun, 君) |
| Corni Fructus (Shanzhuyu, 山茱萸) | 6 | 2 | Minister (Chen, 臣) |
| Moutan Cortex (Mudanpi, 牡丹皮) | 6 | 1.2 |  |
| Pinelliae Rhizoma Praeparatum (Fabanxia, 法半夏) | 10 | 2 |  |
| Salviae Miltiorrhizae Radix et Rhizoma (Danshen, 丹參) | 10 | 2 | Assistant (Zuo, 佐) |
| Bupleuri Radix (Chaihu, 柴胡) | 10 | 2 |  |
| Poria (Fuling, 茯苓) | 10 | 2 |  |
| Chuanxiong Rhizome (Chuanxiong, 川芎) | 6 | 1.2 |  |
| Persicae Semen (Taoren, 桃仁) | 10 | 2 |  |
| Puerariae Lobatae Radix (Gegen, 葛根) | 15 | 3 |  |
| Ziziphi Spinosae Semen (Suanzaoren, 酸棗仁) | 15 | 3 |  |
| Magnetitum (Cishi, 磁石) | 6 | 1.2 |  |
| Margaritifera Concha (Zhenzhumu, 珍珠母) | 10 | 2 |  |
| Dipsaci Radix (Xuduan, 續斷) | 10 | 2 |  |
| Acori Tatarinowii Rhizome (Shichangpu, 石菖蒲) | 6 | 1.2 | Guide (Shi, 使) |
