# Supplementary material for: A Chinese Medicine Formula (Bushen Huoxue Tongluo) for the Treatment of Chronic Subjective Tinnitus: A Study Protocol for a Pilot, Assessor-Blinded, Randomized Clinical Trial
Source: Front Pharmacol. 2022 Mar 30;13:844730. doi: 10.3389/fphar.2022.844730 (PMC9006145; doi:10.3389/fphar.2022.844730)
Supplement: Supplementary file 2 [file Table2.DOCX]

**Table 1 Ingredients of the Chinese herbal formula BHT**

| **Constituent herb** | **Gram/**  **Day, herb** | **Gram/**  **Day, granule** | **Role in formula** |
| --- | --- | --- | --- |
| Rehmannia glutinosa (Gaertn.) DC. [Orobanchaceae] (Dihuang, 地黃) | 12 | 2.4 | Sovereign (Jun, 君) |
| Cornus officinalis Siebold & Zucc. [Cornaceae] (Shanzhuyu, 山茱萸) | 6 | 2 | Minister (Chen, 臣) |
| Paeonia × suffruticosa Andrews [Paeoniaceae] (Mudanpi, 牡丹皮) | 6 | 1.2 |  |
| Pinellia ternata (Thunb.) Makino [Araceae](Fabanxia, 法半夏) | 10 | 2 |  |
| Salvia miltiorrhiza Bunge [Lamiaceae] (Danshen, 丹參) | 10 | 2 | Assistant (Zuo, 佐) |
| Bupleurum falcatum L. [Apiaceae] (Chaihu, 柴胡) | 10 | 2 |  |
| Poria cocos (Schw.) Wolf (Fuling, 茯苓) | 10 | 2 |  |
| Conioselinum anthriscoides 'Chuanxiong' [Apiaceae](Chuanxiong, 川芎) | 6 | 1.2 |  |
| Prunus persica (L.) Batsch [Rosaceae](Taoren, 桃仁) | 10 | 2 |  |
| Pueraria montana var. lobata (Willd.) Maesen & S.M.Almeida ex Sanjappa & Predeep [Fabaceae] (Gegen, 葛根) | 15 | 3 |  |
| Ziziphus jujuba Mill. [Rhamnaceae](Suanzaoren, 酸棗仁) | 15 | 3 |  |
| Magnetitum (Cishi, 磁石) | 6 | 1.2 |  |
| Margaritifera Concha (Zhenzhumu, 珍珠母) | 10 | 2 |  |
| Dipsacus asper Wall. ex DC. [Caprifoliaceae] (Xuduan, 續斷) | 10 | 2 |  |
| Acorus calamus var. angustatus Besser [Acoraceae](Shichangpu, 石菖蒲) | 6 | 1.2 | Guide (Shi, 使) |
